# Mitral Valve Procedures and Multivessel CABG through a Single Left Anterior Minithoracotomy

**DOI:** 10.1055/a-2765-7072

**Published:** 2025-12-15

**Authors:** Volodymyr Demianenko, Hilmar Dörge, Markus Schlömicher, Marius Grossmann, Ahmed Belmenai, Christian Sellin

**Affiliations:** 1 Department of Cardiothoracic Surgery, Heart-Thorax Center, Klinikum Fulda, University Medicine Marburg, Campus Fulda, Fulda, Germany

**Keywords:** minithoracotomy, mitral valve surgery, coronary artery bypass grafting, total coronary revascularization via left anterior thoracotomy, TCRAT

## Abstract

We describe a technique for concomitant coronary artery bypass grafting (CABG) and mitral valve (MV) replacement or annuloplasty with ring implantation performed through a single left anterior minithoracotomy (LAmT). Four patients underwent combined MV and CABG surgery using peripheral cardiopulmonary bypass, a transseptal approach to the MV, and complete coronary revascularization. MV exposure was successfully achieved in all cases without conversion to sternotomy. No major complications such as stroke, reoperation for bleeding, or early mortality occurred. Our initial results demonstrate that single LAmT is a feasible sternum-sparing approach for patients requiring simultaneous coronary and mitral procedures.

## Introduction

Traditional mitral valve (MV) surgery is typically performed via median sternotomy. With advances in minimally invasive cardiac techniques, isolated MV procedures commonly utilize right-sided minithoracotomy. However, combined MV and coronary procedures remain predominantly sternotomy-based due to extensive exposure requirements.

We previously standardized the left anterior minithoracotomy (LAmT) approach for multivessel coronary artery bypass grafting (CABG), known as the Total Coronary Revascularization via left Anterior Thoracotomy (TCRAT) technique. 1 Based on this experience, we explored the feasibility of accessing the aortic valve via the same incision. 2 3 We have now expanded this approach to encompass MV surgery such as annuloplasty with ring implantation or replacement, allowing both MV procedures and complete coronary revascularization through a single minimally invasive incision.

## Technique Description

**Video 1** A detailed step-by-step guide to performing combined mitral valve and coronary artery bypass grafting procedures via a single left anterior minithoracotomy. Step-by-step demonstration of combined mitral valve procedure and multivessel CABG through a single LAmT. Key operative steps include patient positioning, incision, LIMA harvest, CPB setup, coronary anastomoses, transseptal mitral valve exposure, valve procedure, and final graft configuration. CABG, coronary artery bypass grafting; CPB, cardiopulmonary bypass; ICS, intercostal space; LamT, left anterior minithoracotomy; LIMA, left internal mammary artery; MV, mitral valve.


All patients routinely underwent preoperative computed tomography angiography for evaluation of vascular anatomy and screening for significant atherosclerosis or calcifications. Transesophageal echocardiography (TEE) was performed preoperatively to assess MV morphology, function, and the severity of regurgitation.

General anesthesia induction followed an opioid-based multimodal approach. Placement of a jugular venous central line and a jugular venous return cannula, along with systemic heparinization, preceded cardiopulmonary bypass (CPB) cannulation. A 5- to 6-cm-long skin incision was made along the left fourth intercostal space (ICS), starting at the left sternal border. A muscle-sparing dissection was performed, and a rib spreader with concave blades was inserted. The left internal mammary artery (LIMA) was harvested using a pedicled technique with long-tip diathermy, starting from the distal bifurcation and extending proximally beyond the origin of the left mammary vein. CPB was initiated via open Seldinger axillary arterial cannulation and percutaneous venous cannulation with vacuum-assisted venous drainage. 4

**Fig. 1:**
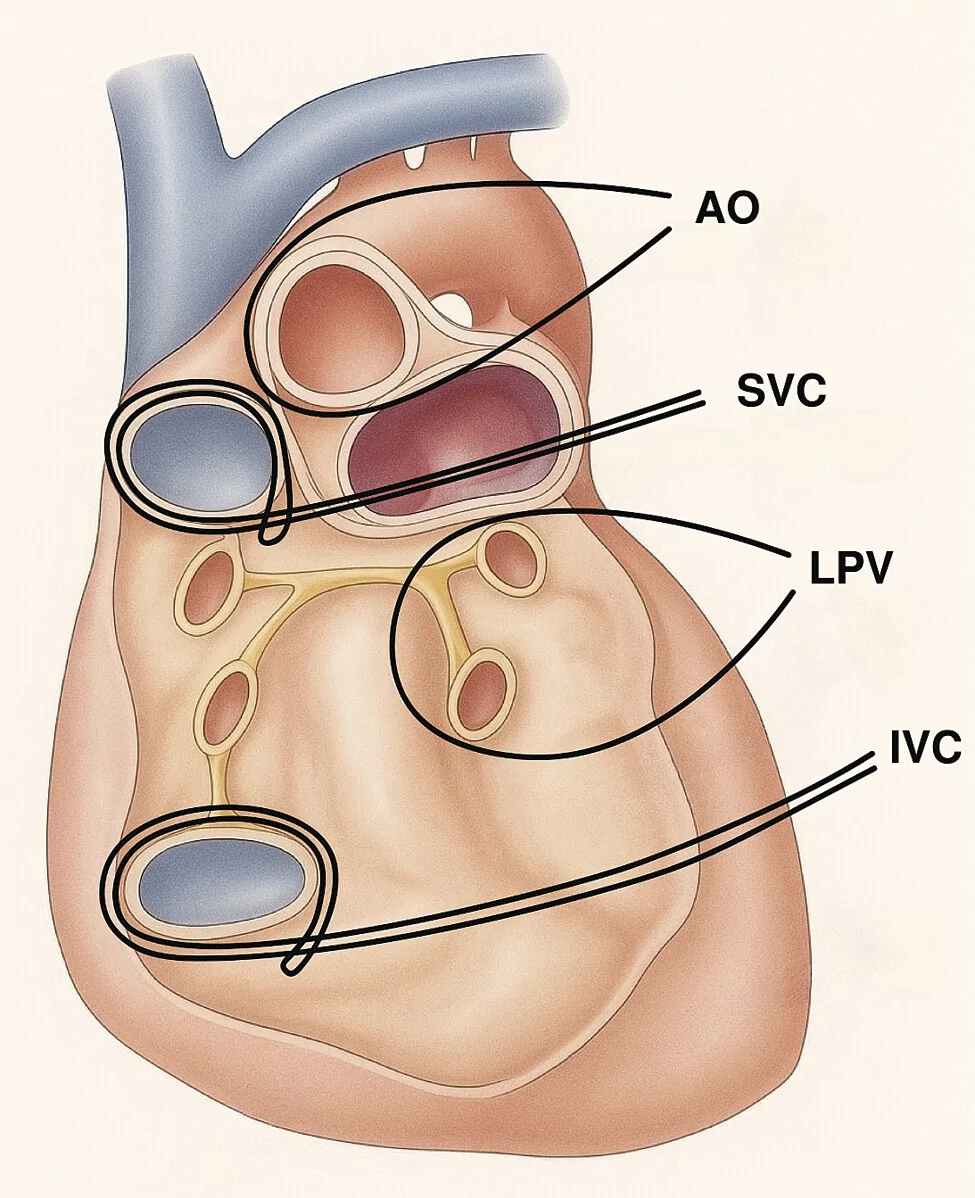
Schematic showing bands placement around the vessels. AO, aorta; IVC, inferior vena cava; LVP, left pulmonary vein; SVC, superior vena cava.

The pericardium was opened in a T-shaped fashion. The ascending aorta was encircled and cross-clamped using a transthoracic clamp introduced through the second ICS. Cold blood cardioplegia was delivered for myocardial protection. Tapes placed around the inferior vena cava, superior vena cava, and left pulmonary veins ( Fig. 1 ) facilitated exposure of the coronary targets and MV.

The first step was coronary revascularization, initiated with distal anastomoses to the lateral and inferior walls. This strategy allowed the grafts to be used subsequently for intermittent cardioplegia delivery.

**Fig. 2:**
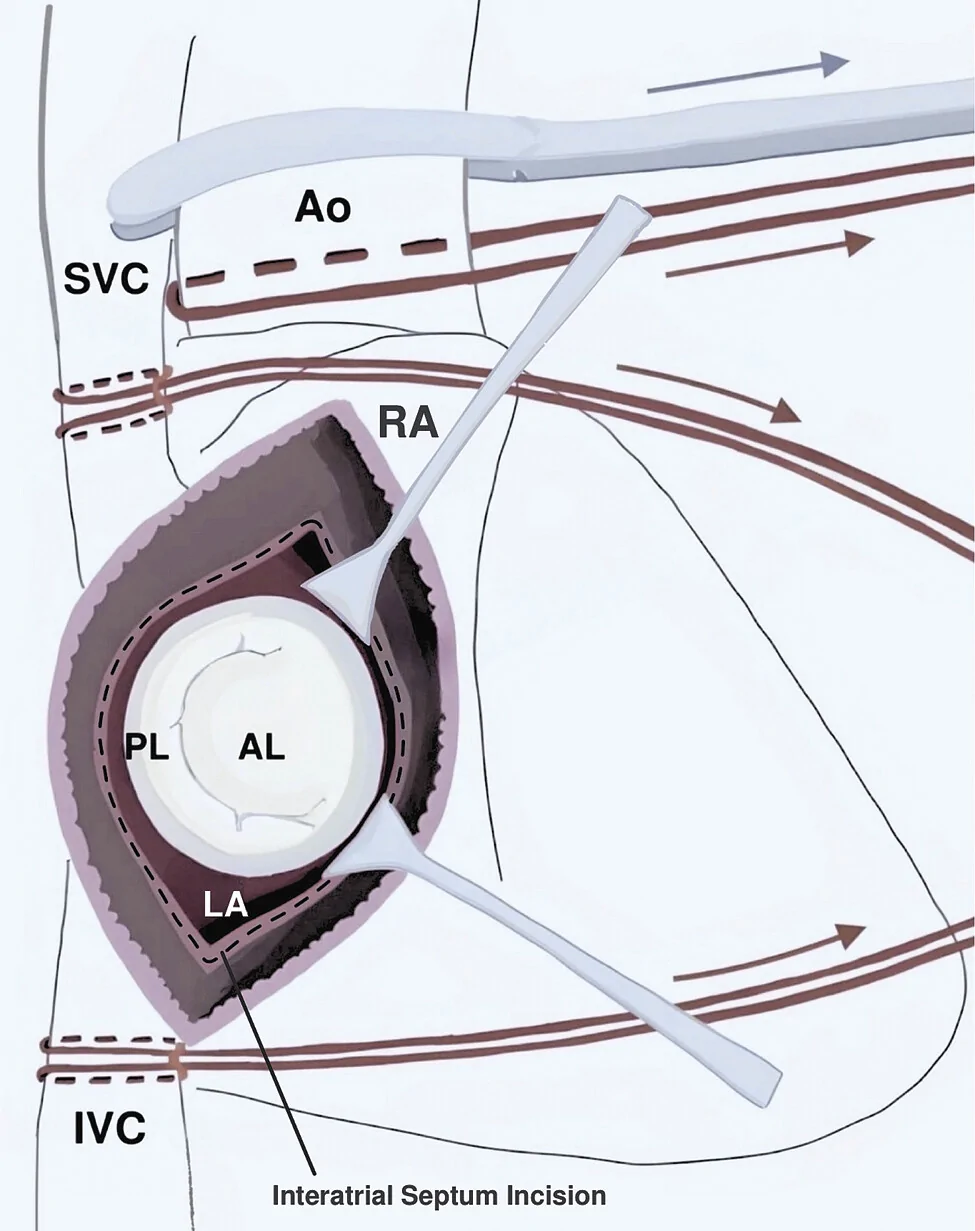
Visualization of mitral valve exposure through interatrial access. Arrows indicate the directions of band tension. AL, anterior leaflet; Ao, aorta; IVC, inferior vena cava; LA, left atrium; PL, posterior leaflet; RA, right atrium; SVC, superior vena cava.

The next step was the MV procedure, for which the surgeon repositioned the patient to the right side. An important technical aspect for achieving optimal MV exposure is the use of lateral traction tapes around the inferior and superior vena cavae and the ascending aorta. This maneuver displaces the mediastinal structures into the left pleural cavity, thereby optimizing surgical access. The right atrium was opened, and the interatrial septum was vertically incised for standard transseptal MV access. Exposure was maintained using stay sutures and small retractors, employing standard or long-shafted instruments as anatomically required ( Fig. 2 ). Depending on preoperative assessment and intraoperative inspection, either MV procedure was performed using standard techniques. Knotting was facilitated using the Cor-Knot automated fastener (LSI Solutions, Victor, NY). The interatrial septum and right atrium were closed ( Video 1 , available in the online version only).

As the final step, the anastomosis between LIMA and the left anterior descending artery, or the construction of T-grafts, was performed. Proximal coronary anastomoses were completed on the beating heart after aortic cross-clamp removal using a side-biting clamp. Perioperative patient characteristics and operative data are summarized in Table 1 .

**Table 1:** Perioperative patient characteristics and operative data

Age	Sex	MR grade	BMI	EF (%)	EuroSCORE II	Operative time, minutes	CPB time, minutes	Aortic cross-clamp time, minutes	Mitral valve procedure	Graft configuration	RBC (unit)	ICU stay (days)	Hospital stay (days)	Postop Echo findings	Postop complications
43	F	Severe	37	55	1.44	343	212	148	MVR mech (29 mm)	Lima to LAD, SVG to LCx	0	1	10	No paravalvular leakage; MPG 4 mm Hg	None
69	M	Severe	31	55	2.16	419	263	185	MVR bio (33 mm)	LIMA to LAD, SVG to LCx	2	7	14	No paravalvular leakage; MPG 4 mm Hg	Neoplasia (on therapy during ICU stay)
77	M	Moderate to Severe	24	45	3.48	342	196	143	MV repair (ring 34 mm)	LIMA to LAD, RA to LCx and RCA	1	2	10	Trivial MI	None
67	M	Severe	30	50	2.27	315	185	132	MV repair (ring 32 mm)	LIMA to LAD, RA to LCx and RCA	1	2	9	Trivial MI	None

Abbreviations: BMI, body mass index; CPB, cardiopulmonary bypass; EF, ejection fraction; ICU, intensive care unit; LAD, left anterior descending artery; LCx, left circumflex artery; LIMA, left internal mammary artery; MI, mitral insufficiency; MPG, mean pressure gradient; MR, mitral regurgitation; MVR, mitral valve replacement; MV, mitral valve; RA, radial artery; RBC, red blood cell; RCA, right coronary artery; SVG, saphenous vein graft.

## Discussion

Our preliminary experience demonstrates the feasibility of performing combined MV surgery and complete anatomical coronary revascularization through a single LAmT incision. Avoiding sternotomy eliminates the risk of sternal wound complications and accelerates postoperative recovery. Previously described sternum-sparing techniques for combined procedures include bilateral thoracotomies, hybrid methods, or complex transapical approaches. 5 6 The presented technique employs a single incision and standard surgical instruments, thereby streamlining the procedure and enhancing its general applicability.

As TCRAT is an on-pump, arrested-heart procedure, it inherently offers the possibility of combining minimally invasive multivessel CABG with other cardiac procedures. A key element of the presented technique is the use of CPB with cardiac arrest during all surgical steps, which allows controlled manipulation of the decompressed heart as well as safe access to all cardiac structures and optimal exposure within the limited operative field. Despite prolonged cross-clamp times, meticulous myocardial protection using repeated cold blood cardioplegia administered into the aortic root and subsequently through the distal anastomoses prevented myocardial injury and renal dysfunction.

This approach is not suitable for all patients. Exclusion criteria include ascending aortic calcifications, previous cardiac surgery, and complex chest wall anatomy, such as pectus excavatum. At the current stage of our experience, we would not recommend this approach in patients with severe mitral annular calcification or in patients requiring more complex MV repair techniques other than annuloplasty. However, future refinements may represent an avenue for expansion of this approach. Prior experience with the TCRAT technique significantly enhances procedural safety and success.
